# Participating in innovative medicines initiative funded neurodegenerative disorder projects—An impact analysis conducted as part of the NEURONET project

**DOI:** 10.3389/fneur.2023.1140722

**Published:** 2023-03-16

**Authors:** Claire Hawksworth, Fatima Salih, Katharine Cresswell, Lennert Steukers, Carlos Diaz, Lewis Killin, Laurent Pradier, Angela Bradshaw, Dalia Dawoud

**Affiliations:** ^1^Department of Science, Evidence and Analytics, National Institute for Health and Care Excellence, Manchester, United Kingdom; ^2^Department of Science, Evidence and Analytics, National Institute for Health and Care Excellence, London, United Kingdom; ^3^Janssen Pharmaceuticals NV, Beerse, Belgium; ^4^SYNAPSE Research Management Partners, Barcelona, Spain; ^5^Department of Scientific Strategy and External Relations, Sanofi, Paris, France; ^6^Alzheimer Europe, Luxembourg, Luxembourg; ^7^Faculty of Pharmacy, Cairo University, Cairo, Egypt

**Keywords:** Innovative Medicines Initiative (IMI), neurodegenerative disease, impact, survey, public-private partnerships (PPPs), neurodegenerative disorder

## Abstract

The European Commission's Innovative Medicines Initiative (IMI) has funded many projects focusing on neurodegenerative disorders (ND) that aimed to improve the diagnosis, prevention, treatment and understanding of NDs. To facilitate collaboration across this project portfolio, the IMI funded the “NEURONET” project between March 2019 and August 2022 with the aim of connecting these projects and promoting synergies, enhancing the visibility of their findings, understanding the impact of the IMI funding and identifying research gaps that warrant more/new funding. The IMI ND portfolio currently includes 20 projects consisting of 270 partner organizations across 25 countries. The NEURONET project conducted an impact analysis to assess the scientific and socio-economic impact of the IMI ND portfolio. This was to better understand the perceived areas of impact from those directly involved in the projects. The impact analysis was conducted in two stages: an initial stage developed the scope of the project, defined the impact indicators and measures to be used. A second stage designed and administered the survey amongst partners from European Federation of Pharmaceutical Industries and Associations (EFPIA) organizations and other partners (hereafter, referred to as “non-EFPIA” organizations). Responses were analyzed according to areas of impact: organizational, economic, capacity building, collaborations and networking, individual, scientific, policy, patient, societal and public health impact. Involvement in the IMI ND projects led to organizational impact, and increased networking, collaboration and partnerships. The key perceived disadvantage to project participation was the administrative burden. These results were true for both EFPIA and non-EFPIA respondents. The impact for individual, policy, patients and public health was less clear with people reporting both high and low impact. Overall, there was broad alignment between EFPIA and non-EFPIA participants' responses apart from for awareness of project assets, as part of scientific impact, which appeared to be slightly higher among non-EFPIA respondents. These results identified clear areas of impact and those that require improvement. Areas to focus on include promoting asset awareness, establishing the impact of the IMI ND projects on research and development, ensuring meaningful patient involvement in these public-private partnership projects and reducing the administrative burden associated with participation in them.

## 1. Introduction

The Innovative Medicines Initiative (IMI), which has recently been succeeded by the Innovative Health Initiative (IHI) was the world's largest public-private partnership (PPP) in the life sciences. The IMI was a partnership between the European Union (EU), represented by the European Commission, and the European pharmaceutical industry, represented by the European Federation of Pharmaceutical Industries and Associations (EFPIA). EFPIA aims to help members collaborate, innovate and discover new therapies for people across Europe and its members include 37 national associations, 38 pharmaceutical companies and a growing number of small and medium-sized enterprises.

The IMI's core mission was to ‘translate health research and innovation into tangible benefits for patients and society and ensure that Europe remains at the cutting edge of interdisciplinary, sustainable, patient-centric health research'. The IMI achieved this through funding over 159 projects since its launch in 2007 followed by launch of the IMI2 from 2014 to 2020. To give an idea of funding amount, the current total budget for its successor IHI is €2.4 billion with approximately half each coming from Horizon Europe and IHI industry partners, and €200 million coming from other life science industries.

IMI2 funded research that aligned with its Strategic Research Agenda (SRA) (1). This laid out the key disease area and research priorities which governed its funding calls. Another initiative specifically relevant in the neurodegeneration disease space, and to this publication, is the EU Joint Programme- Neurodegenerative Disease Research (JPND). This is the largest global research initiative aimed at tackling the challenge of neurodegenerative diseases and in 2019 it published its Research and Innovation Strategy (2) outlining thematic priorities for future research in order to improve prevention, diagnosis, treatment and patient care for neurodegenerative diseases. IMI projects are partnerships between members of EFPIA and other organizations including academic institutions and small and medium sized enterprises (SMEs).

NEURONET was a 3-year Coordination and Support Action that received nearly €2 million in funding through IMI2. It provided coordination and support to other IMI funded neurodegenerative disorder research projects. It aimed to identify research gaps, communicate research findings and create links between the projects that form the IMI neurodegenerative disorders (ND) portfolio. This portfolio currently includes more than 20 different research projects which are improving the diagnosis, prevention, treatment and understanding of neurodegenerative conditions.

A potential benefit of PPPs is that greater transparency at the pre-competitive stage, and in research and development (R&D) could reduce redundancy, duplication of effort, and save money (3). It is assumed too, that spending on R&D will improve innovation and therefore the IMI ND portfolio should generate innovation in the NDD space. The NEURONET project was tasked with investigating this. Logically, this required an impact assessment which needed to establish which factors likely facilitated pharmaceutical innovation (aligned with the SRA mission and priorities) and how the IMI ND portfolio contributed to these factors.

Impact assessments aim to evaluate the significance and reach of both positive and negative effects of research (4). The definition of impact in the context of NEURONET is restricted due to the lack of baseline to assess change. Impact was therefore evaluated in terms of process and activity in relation to the key principles underlining IMI's objectives. In this impact assessment there were two stages. Stage one characterized the project portfolio and conducted network and publication analyses to understand key organizations involved, the degree of collaboration and how the publications addressed ND research priorities. To understand the latter, the SRA priorities for neurodegenerative disease were mapped against themes from the JPND Research and Innovation Framework and the broader JPND report (2).

The network analysis revealed that EFPIA companies are key vehicles for dissemination of knowledge generated between projects due to their prominent feature in the network (5). For example, they were more likely to work on multiple IMI ND projects, connecting them to more organizations. On the whole academic organizations were underrepresented as these ‘key nodes' in the network. However, the publication analysis revealed that many were authored by single academic institutions or multiple collaborations between academic partners, a finding that has previously been reported in PPPs (6). The authors concluded that further research was needed to understand if this limited cross-public-private partner collaboration on publications is reflective of an overall lack of collaboration across organizations, or if collaboration across organizations is demonstrated through other mechanisms such as the development of project assets. The publication analysis also revealed a need to more broadly assess how project assets are contributing toward research across the priority scientific areas.

Overall, the first stage provided NEURONET with interesting findings on collaboration, networking and research impact that warranted more in-depth analysis, as well as a broader assessment of impact to align with IMI's objectives. Themes that the second stage was to explore included:

- Reasons for single organization publications and impact this has on knowledge generation and transfer between organizations. Further work could also explore why certain organizations do not participate in publications and whether or not this hinders the transfer of knowledge;
- How the IMI portfolio is linking to global research efforts in this field;
- Qualitative research looking more broadly at the use and impact of project assets, particularly by EFPIA;
- The impact on EFPIA companies of collaborations with other partners through IMI projects;
- Exploration of impact on personal and professional development and the creation of opportunities for early career researchers.

These informed the design of a survey for partners who were involved in the IMI ND projects to understand the broader impact of the projects. The scope for the survey therefore became to understand the scientific and socio-economic impact of the IMI ND portfolio across the EU. Recently various frameworks to measure the impact and value of PPPs have been proposed, and all recognize that wider measures of impact are needed to appropriately reflect their value (7–9). To operationalize scientific and socio-economic impact, we fractioned it into key areas of impact that together would provide insight into the wider impact of the IMI ND portfolio. These areas of impact were: organizational, economic, capacity, collaboration, individual, scientific, policy, patient, societal and health impacts. The survey questions were organized around these key themes.

EFPIA companies were initially targeted for the survey due to the findings from stage one that they represented key organizations in the IMI ND portfolio. After conducting this exercise it was felt that it would be valuable to repeat it for the other organizations involved in the projects which included academic organizations, SMEs patient/carer organizations and other organization types. These could be termed “research-related organizations” and we refer to these as “non-EFPIA” organizations in this publication. Repeating the survey with this group allowed insight into the impact of the IMI ND portfolio from all perspectives.

This paper reports on the conduct and results of the surveys to illustrate the range of project impacts.

## 2. Methods

### 2.1. Data collection

To traditionally evaluate impact there needs to be a baseline in which to assess change. The definition of impact in the context of NEURONET is restricted due to the lack of baseline. In addition, NEURONET is not acting as an auditor or evaluator of individual projects or the impact of any specific deliverables against the projects aims. Impact was therefore evaluated in terms of process and activity in relation to the key principles underlining IMI's objectives.

At the time of the survey there were 18 projects in the ND portfolio. See Supplementary Table 1 for the full list. Seven had completed, four were coming to an end and seven were ongoing. The survey was the second stage of the impact analysis. The first stage characterized the project portfolio. For each project data was collected on the partner organizations and number of assets. These were used to conduct a network analysis which visualized the IMI ND portfolio. Every unique organization represented a node and connections between the nodes were defined by the IMI projects in common. Measures of centrality were calculated including the “degree” and “betweenness” of all the network nodes. The degree gave the number of ties that one organization has to all other organizations in the network and the betweenness represented the number of times a node is present in the shortest path between two nodes. This was conducted in Rstudio.

The publication analysis in the first stage of the impact assessment included eight projects that had completed or were about to finish their activity. The following information on the project publications were collected: title, Digital Object Identifier (DOI), first author, first author organization, organizations of all co-authors on the publication. The methodology followed that used by IMI for its annual bibliographical analysis of all IMI projects (10). The number of project organizations for each publication was calculated along with the number of publications per project, the number and percentage of project organizations on at least 1 publication, the number and mean number of publications per organization and the percentage of all project publications each organization was listed on. Two hundred and thirty two publications were included. A network analysis was conducted following the methods and measures for the project network analysis. This indicated how collaborative organizations were. A framework was developed to analyze the project publications against key ND scientific priorities. To do this, the SRA priorities were mapped to the overarching themes from the JPND Research and Innovation Framework, in addition to a number of other sub-categories identified from the JPND report. A visual heatmap was created using MS Excel to show the research priorities and the extent to which these were being addressed. Full details for the methods used in stage one of the impact analysis are documented in the final report (11) and have been published (5).

Stage two of the impact assessment involved developing a survey to elicit more detail on areas of further research generated from stage one, along with data on the broader impact of the IMI ND portfolio. A questionnaire for the EFPIA organizations was developed by Janssen, Belgium, as Task Lead and refined following input from members of the NEURONET Executive Committee (ExCom). Work Package 1 Lead, the National Institute of Health and Care Excellence (NICE), UK, piloted the survey to check face validity and time for completion.

The survey was divided into six categories, informed by stage one of the impact assessment: experience in IMI, impact on company, impact on daily work, impact on professional career, impact on professional network and impact on the field at large. The survey had 46 questions.

The survey was administered online and disseminated between 29th March and 31st August 2021. All EFPIA partners' staff that are or have been involved in one or more of the 18 IMI ND projects that were part of the portfolio at the time of the survey were invited to complete it. The survey was distributed through the companies *via* a named NEURONET contact person and/or the IMI operational contact person of each EFPIA company. To increase response rates from individual companies a final reminder was sent by the IMI scientific officer on 13 August 2021.

After the EFPIA survey was closed, it was felt that it would be valuable to also survey other project organizations or “non-EFPIA” organizations. The EFPIA survey was reviewed and adapted by a multi-disciplinary group including “non-EFPIA” representation to ensure questions were relevant for a non-EFPIA audience, and therefore facilitate responses. The group included considerations such as the role of the stakeholders, funders or research managers, or executers, the structure of the organization, and terminology and traditional measures of impact in different sectors e.g., publications in academia. This removed questions on economic and regulatory and policy impact. The final survey was drafted by NICE, UK, and refined following input from the NEURONET ExCom. NICE, UK, piloted the survey to check face validity and time for completion. The survey was divided into six categories: experience in IMI, impact on research group or department or personnel, impact on research, impact on collaborations, broader impact on society, research and innovation and impact of assets. The survey had 21 questions.

The survey was disseminated to the individual portfolio projects' partners through their project leads, and through project managers of individual projects. The online survey for non-EFPIA partners was administered between January and March 2022. The online survey tool, Survio^®^, was used for both surveys.

See Supplementary Tables 2, 3 for the EFPIA and non-EFPIA surveys, respectively.

A pragmatic search was carried out using a pearl growing strategy. Research Rabbit and CitationChaser were used to identify references related (by citation or topic similarity) to known, relevant records which were found by searching PubMed and Google Scholar with search terms including “Innovative Medicines initiative,” “impact assessment” and “neurodegenerative disorders.”

### 2.2. Data analysis

The EFPIA survey questions were categorized into 10 areas of impact for data analysis purposes. See Supplementary Table 4 for how they were categorized. These outcome categories were pre-defined and originated from a different task within NEURONET that was developing complementary Key Performance Indicators (KPIs) to estimate impact. These were based on existing IMI KPIs, the IMI1 impact assessment reports and discussions with EFPIA representatives within NEURONET. The outcome areas of impact were:

- Organizational impact (e.g., organizational strategy, objectives, planning, processes, reputation etc.).
- Economic impact (e.g., return on investment).
- Capacity building (e.g., professional development, attracting new staff).
- Collaborations, networks and partnerships.
- Individual impact (e.g., personal development, collaborations and networks, ways of working).
- Scientific impact (e.g., impact on the drug development process, awareness & visibility of IMI ND projects/assets and use of assets in R&D and regulatory/HTA practice).
- Regulatory and policy impact (e.g., impact on regulatory practice, decision makers).
- Patient impact (e.g., research that is including patients and bringing science closer to them).
- Societal impact (e.g., research that is including and empowering the public and generating outcomes and impacts that are relevant for patients/citizens).
- Health impacts (impacts on public health, e.g., life expectancy, prevention of illnesses, quality of life, and the health-care system).

For the non-EFPIA survey, the results were categorized and analyzed according to the seven areas of impact deemed most relevant:

- Organizational impact.
- Collaborations, networks and partnerships.
- Individual impact.
- Scientific impact.
- Patient impact.
- Societal impact.
- Health impacts.

The responses to the survey were exported from Survio to Excel and analyzed. Quantitative and qualitative data were collected. Quantitative data were analyzed using descriptive statistics (counts and percentages of different responses) and responses to the open-ended questions were coded and thematically analyzed using an inductive approach.

## 3. Results

### 3.1. Survey respondents

#### 3.1.1. EFPIA

Overall, for the EFPIA survey, 91 responses were received from 24 out of the 31 companies that were invited to participate. See Supplementary Table 5 for the full list of the 24 companies. The majority of responses were from Janssen Pharmaceutica NV and Sanofi (57%, *n* = 49/86). Five respondents indicated that they were not involved in any IMI project and did not qualify for inclusion. The final analysis included 86 responses.

On average, the EFPIA respondents were involved in 2 projects with the majority spending at least 2 hours per week on the projects (74%, *n* = 64/86). Nearly half of those (47%, *n* = 30/64) spent more than 6 h per week on the projects. In terms of project role, the majority (64%, *n* = 55/86) had not been Project Lead (i.e., responsible for the delivery of the whole project) on any project while half of the respondents (50%, *n* = 43/86) indicated they had been Work Package Lead (i.e., responsible for the delivery of the activities of a single work package) on at least 1 project.

#### 3.1.2. Non-EFPIA

**Figure 1:**
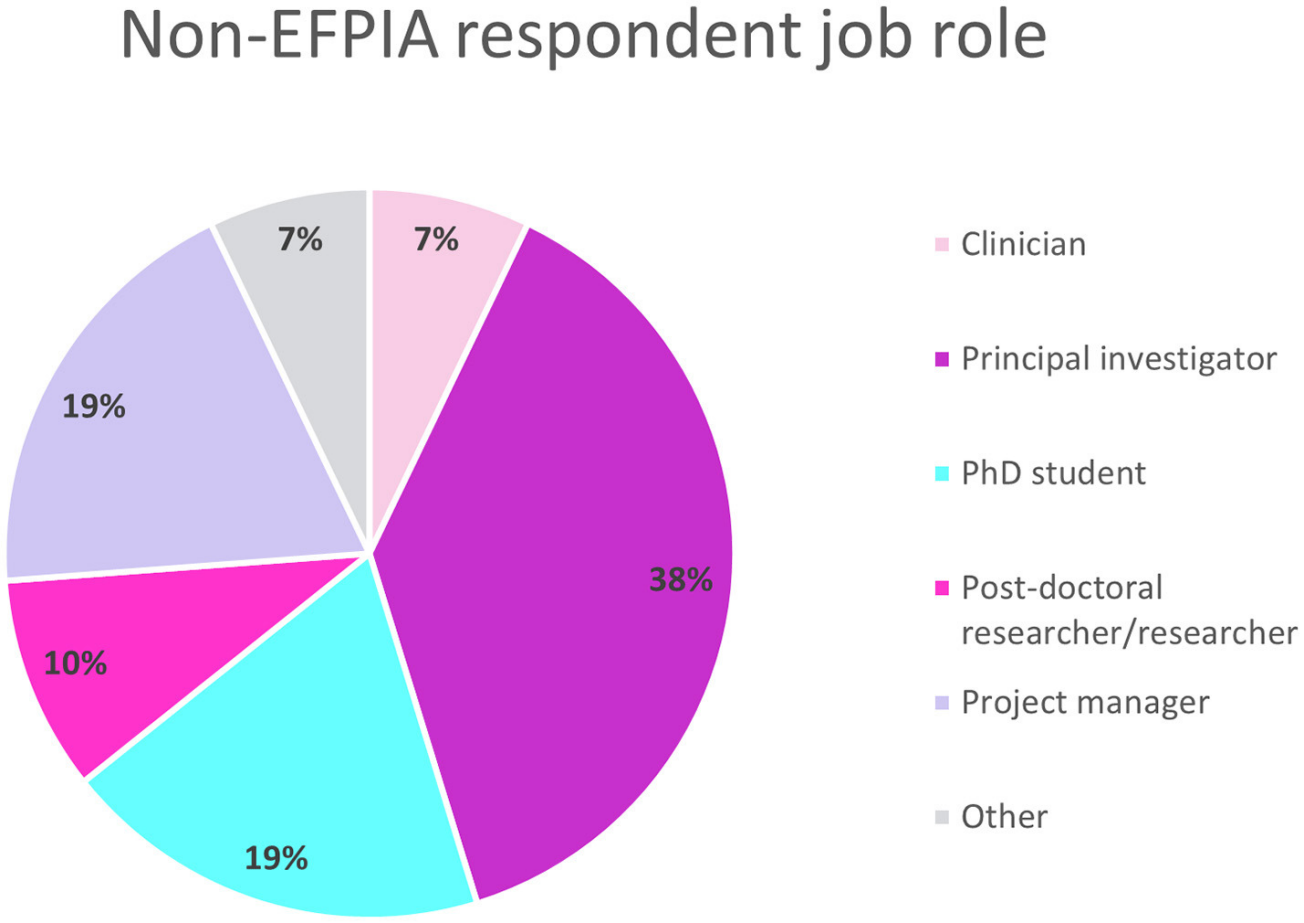
Non-EFPIA survey question assessing respondents job role. Respondents selected from Principal Investigator, post-doctoral researcher, clinician, PhD student, project manager, technician, or other. *N* = 42.

Overall, 43 people completed the survey, however one was from an EFPIA organization and excluded from the analyses. The final analysis included 42 respondents. The respondents had roles ranging from Principal Investigator to post-doctoral researchers and project managers (Figure 1). The respondents were split equally between spending 5–10%, 10–50% or more than 50% of their time on the IMI projects.

### 3.2. Organizational impact

#### 3.2.1. EFPIA

The responses confirmed the visibility of IMI projects within EFPIA organizations. The IMI was known within companies for 100% of respondents and 58% of respondents (*n* = 50/86) thought there were aspects of R&D done differently due to IMI projects.

Over a third of respondents (37%, *n* = 32/86) thought the project they were involved in had a “moderate or high” impact on the company's strategic objectives and ways of working overall, although an equal proportion also thought the impact on this had been “neutral.” The majority of respondents (65%, *n* = 56/86) also thought that the IMI ND projects had an impact on the company's presence, visibility and public perception.

Although “I don't know”' was the most popular answer when asked whether the company helps in creating awareness of project outcomes (43%, *n* = 37/86), or helps in creating awareness of the impact of those outcomes (44%, *n* = 38/86), of the remaining respondents, more answered “yes” than “no” to these questions (38 and 35% vs. 19 and 21%, respectively).

#### 3.2.2. Non-EFPIA

Nearly all respondents (88%, *n* = 37/42) felt the projects had resulted in a change to their department. Most thought a “slight” change (45%, *n* = 19/42), followed by a ‘moderate' (33%, *n* = 14/42) and “radical” change (10%, *n* = 4/42).

The majority of respondents reported an expansion to current research lines (62%, *n* = 26/42). Nearly half of respondents reported that involvement had led to the creation of new research lines (45%, *n* = 19/42) and an improvement in global positioning (43%, *n* = 18/42). Over a third (38%, *n* = 16/42) also saw new contracts or funding opportunities in their organization due to involvement in IMI ND projects. Other organizational impacts described by respondents include being able to finance staff locally and diversify the staff involved in projects.

### 3.3. Economic impact

**Figure 2:**
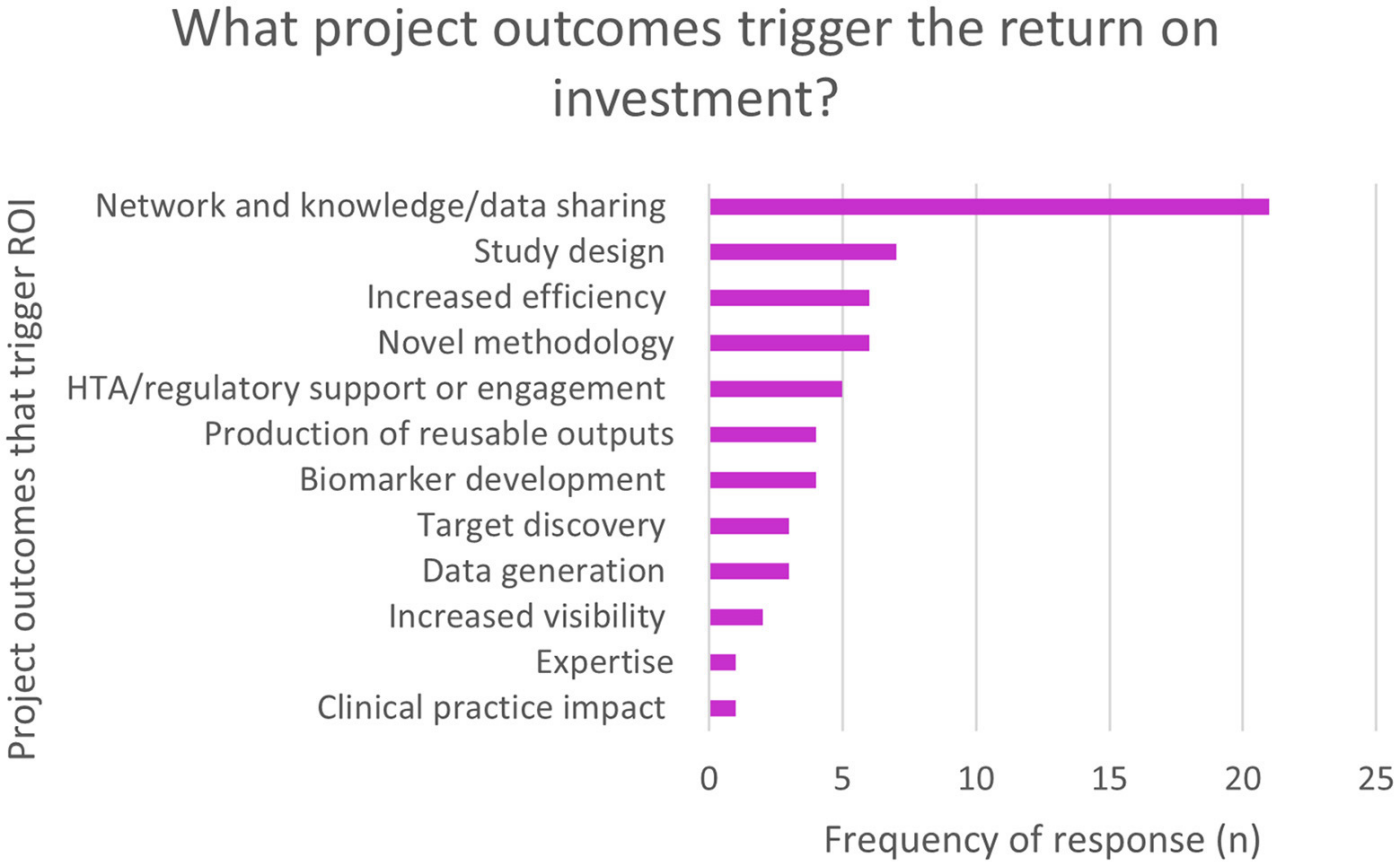
EFPIA survey question asking about economic impact. Respondents rated the return on investment in terms of increased efficiency, acceleration of processes, new knowledge etc., and were then asked to elaborate on what project outcomes triggered the return on investment. This figure shows these themes. *N* = 56 for respondent who used free text to detail the project outcomes.

Economic impact was only assessed in the EFPIA survey. Overall, 50% (*n* = 43/86) of respondents selected “neutral” when asked about the impact of the projects on return on investment (ROI). The survey prompted respondents to elaborate on which project outcomes triggered the ROI. Figure 2 shows that the major outcomes were around networking, knowledge and data sharing.

## 3.4. Capacity building

### 3.4.1. EFPIA

**Figure 3:**
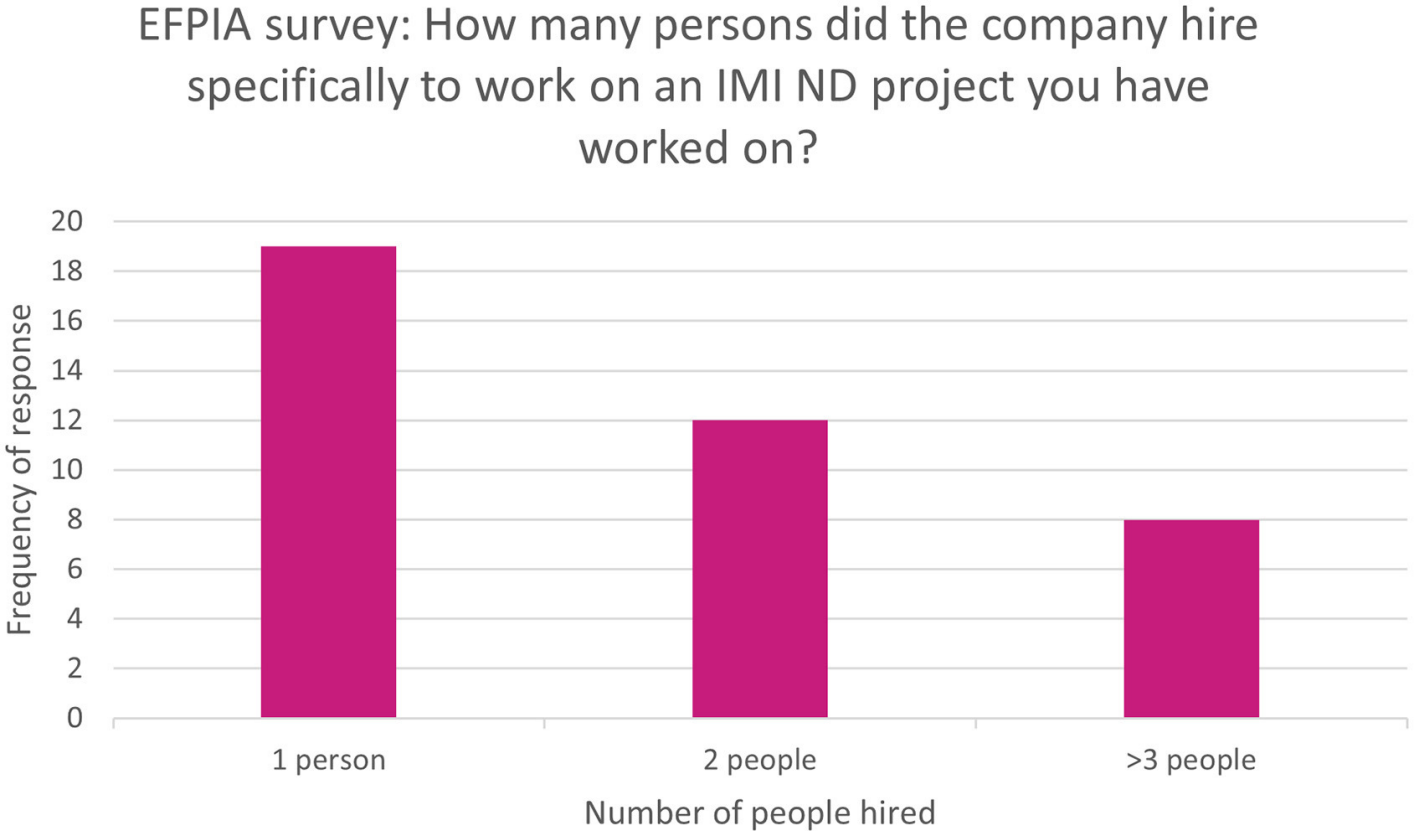
EFPIA survey question asking about number of new recruits to IMI ND projects. Respondents were asked “how many persons did the company hire specifically to work on an IMI ND project you have worked on?” *N* = 39 respondents who indicated there had been hires specifically for the IMI ND project. These were grouped into 1, 2, or more than 3.

Of all respondents, 41% (*n* = 36/86) rated the impact on attracting talent as moderate or high. Additionally, 45% (*n* = 39/86) of respondents reported that people had been hired specifically to work on the IMI ND project. The breakdown of the number of hires is shown in Figure 3. Nearly half of those who reported hires (49%, *n* = 19/39) were aware of people who went on to receive a permanent position after being hired for an IMI ND project. Furthermore, 12% (*n* = 10/86) of respondents were aware of people who were hired from an IMI ND project partner to their company.

### 3.4.2. Non-EFPIA

The majority of respondents reported an increase in the number of staff (64%, *n* = 27/42) due to involvement in the IMI ND project.

## 3.5. Collaborations, networks, and partnerships

### 3.5.1. EFPIA

Nearly half of respondents (49%, *n* = 42/86) rated the projects' impact on establishing strategic partnerships as “moderate” or “high.” “I don't know” was the most popular answer (53%, *n* = 46/86) when asked if the respondents were aware of any strategic partnerships formed between the company and other IMI partners. Of the remaining, slightly more said yes (26%, *n* = 22/86) than no (20%, *n* = 17/86).

Most people (81%, *n* = 70/86) did report meeting new people internally at their own company and 93% (*n* = 80/86) reported meeting new people from different companies. Around 78% (*n* = 67/86) of respondents also reported establishing new long-term relationships with academic institutions, SMEs, Biotechs and patient organizations and of those 67% (*n* = 45/67) reported forming one to five new long-term relationships.

### 3.5.2. Non-EFPIA

**Figure 4:**
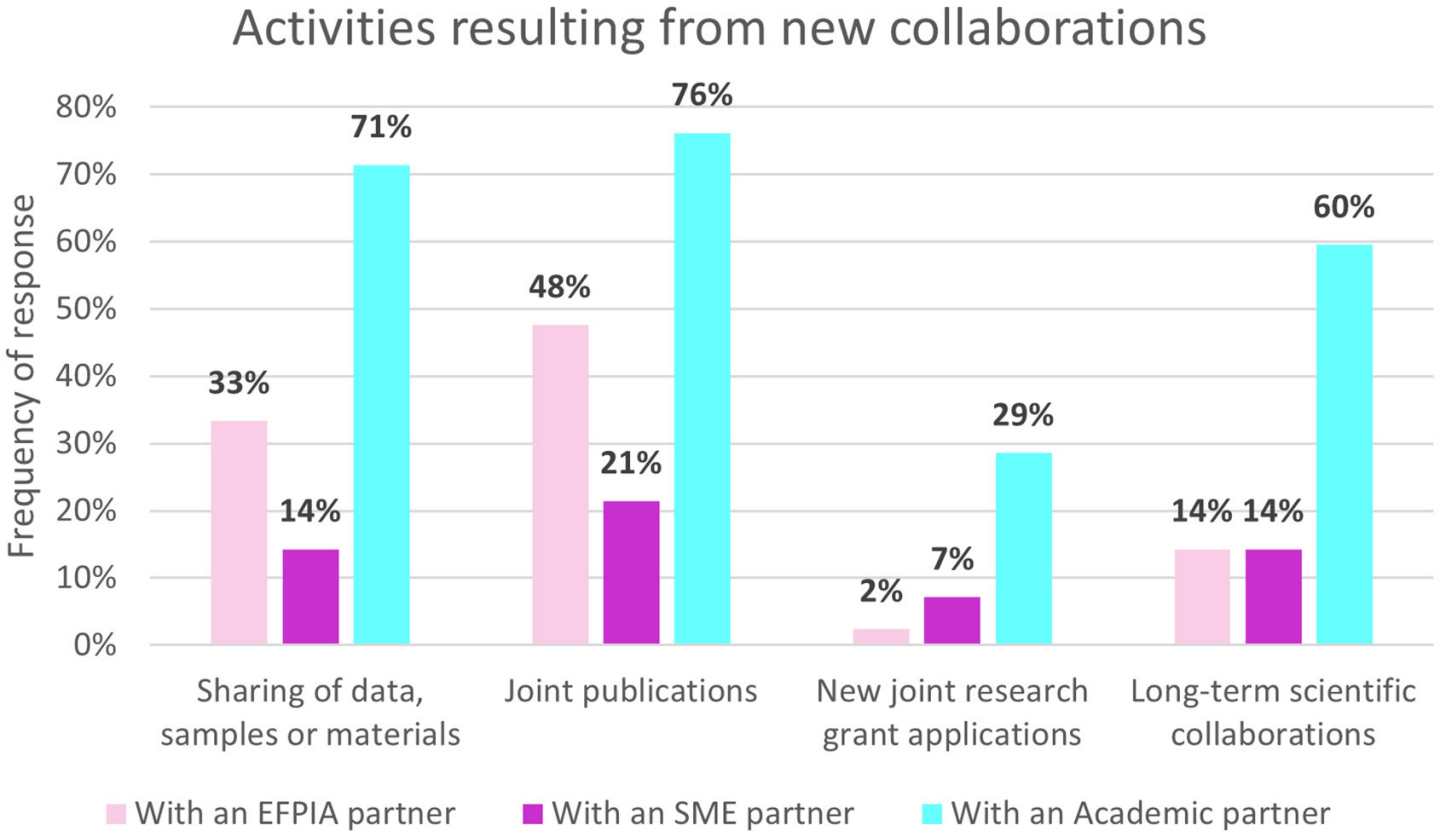
Non-EFPIA survey question assessing new collaborations. Respondents were asked “did these new collaborations result in” and could select from: sharing of data, samples or materials, joint publications, new joint research grant applications, long-term scientific collaborations. Respondents indicated whether these were: with an EFPIA partner, an SME partner, academic partner or there was no type of collaboration. *N* = 42.

All respondents reported meeting new people from other organizations and nearly half also met new people in their own organizations (43%, *n* = 18/42). Some of the impacts resulting from these new connections are presented in Figure 4. The most common type of collaboration arising from these connections has been with academic partners, followed by EFPIA partners and then SMEs. Sharing of data and joint publications with academic partners were the most frequently stated activities with academic partners (71%, *n* = 30/42 and 76%, *n* = 32/42 respectively). A third of respondents (31%, *n* = 13/42) had interacted with a regulatory or health technology assessment (HTA) body in relation to IMI ND research as a direct result of participation in IMI projects.

## 3.6. Individual impact

### 3.6.1. EFPIA

**Figure 5:**
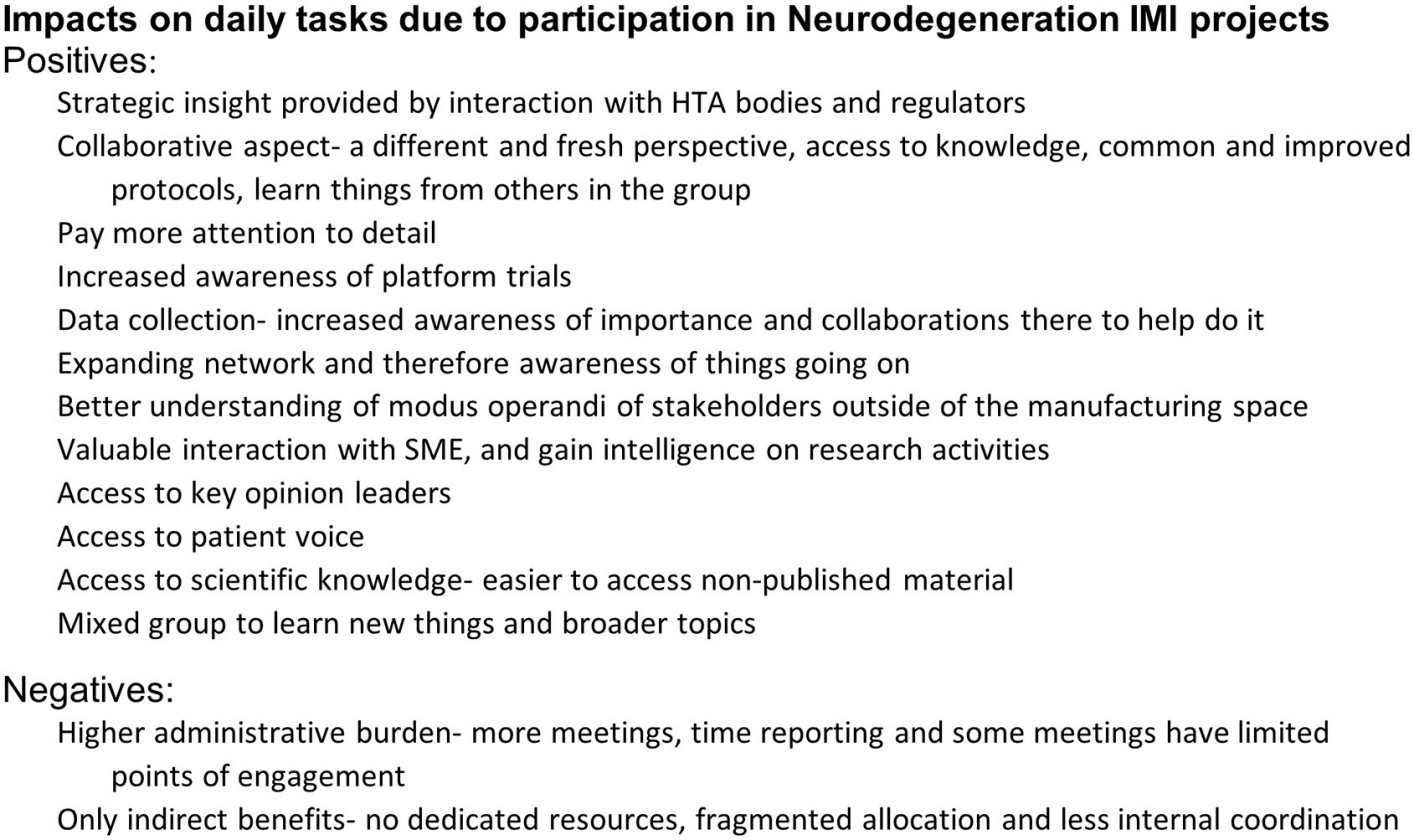
EFPIA survey question assessing the impact on daily tasks. Respondents were asked to rate the impact of IMI on how they perform their daily tasks and could then highlight any of these impacts using a free text box. These themes are summarized in this figure. *N* = 31 respondents who highlighted specific impacts.

The reported degree of impact on individuals' daily tasks varied, where 30% (*n* = 26/86) chose “some” or “high” impact and 21% (*n* = 18/86) chose ‘no impact' while the remainder were neutral. Overall, 38% (*n* = 33/86) said they do use new tools/datasets/knowledge generated through an IMI project in their daily work whilst 48% (*n* = 41/86) said they did not and 14% (*n* = 12/86) said “I don't know.” Some respondents (36%, *n* = 31/86) detailed the impacts on their daily tasks. These are summarized in Figure 5.

In terms of available support, 76% (*n* = 65/86) of respondents said they have or had support from their managers to work on IMI projects while 7% (*n* = 6/86) commented “supportive in theory but no resource commitment or adjustment to other deliverables.” Additionally, 60% (*n* = 52/86) of respondents said they received appreciation from their employer for working on IMI projects. In terms of resources, 48% (*n* = 41/86) of respondents said they did have sufficient resources and time to fulfill their assigned tasks, 44% (*n* = 38/86) who said they were not sufficiently resourced.

Overall, 81% (*n* = 70/86) of respondents detailed how IMI had impacted their skillset as shown in Table 1. Furthermore, 81% (*n* = 70/86) agreed that IMI had expanded their scientific horizons and of these 76% (*n* = 53/70) specified how, as detailed in Table 2.

**Table 1:** Themes and their frequencies when asked how IMI projects had impacted skill set.

**Theme describing how IMI has improved skill set**	Frequency (%)^*^
Collaboration for problem solving/networking/communicating externally/project management	46 (66%)
Improved understanding of neurodegenerative disease field/current data and issues	17 (24%)
Knowledge of and access to new techniques/tools/data analytical methods	13 (19%)
Understanding of current research activities	1 (1%)
Not applicable	16 (23%)

^*^N = 70 respondents. Some respondents gave multiple responses.

**Table 2:** Themes and their frequencies when asked how participation in IMI projects had expanded scientific horizons.

**Theme describing how IMI has expanded horizons**	Frequency (%)^*^
Broader perspective and understanding alternative approaches by interacting with external colleagues	19 (36%)
Understanding research landscape	14 (26%)
Learning from experts in the field	9 (17%)
Expanding network	7 (13%)
Exposure and access to novel research techniques and technologies	5 (9%)
Knowledge of unpublished data	4 (8%)
Interaction with academic partners	1 (2%)

^*^N = 53 respondents who detailed how. Some respondents gave multiple themes.

When all respondents were asked if any new opportunities came their way directly or indirectly through participation in an IMI project, 53% (*n* = 46/86) responded with “no,” 35% (*n* = 30/86) responded with “yes” and 12% (*n* = 10/86) responded with “I don't know.”

### 3.6.2. Non-EFPIA

**Figure 6:**
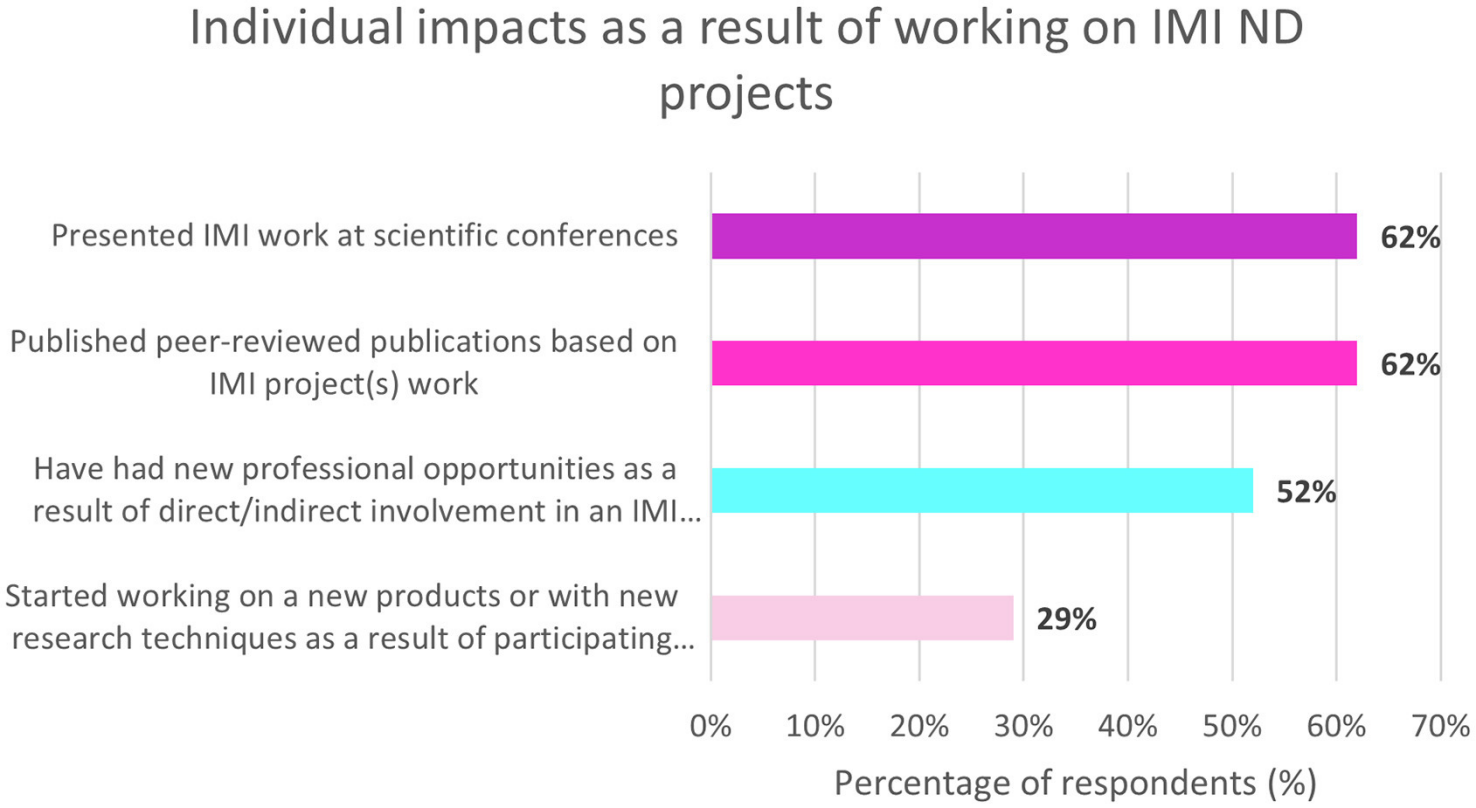
Non-EFPIA survey questions assessing the impact on individuals. Respondents were asked four questions: “did any new professional opportunities come your way directly/indirectly through participation in an IMI project?” (yes/no), “have you started working on any new products or with new research techniques as a result of participating in IMI projects?” (yes/no), “have you published any peer-reviewed publications based on your work in IMI projects?” (yes/no) and “did you present any of your IMI project work at scientific conferences?” (yes/no). *N* = 42.

On the other hand, 69% (*n* = 29/42) of non-EFPIA respondents felt that involvement in IMI ND projects has resulted in a beneficial impact on their career. The impacts on individuals working on IMI ND projects are shown in Figure 6. These included presenting at scientific conferences and publishing peer-reviewed publications.

Qualitative findings suggest that the benefit of being involved in an IMI ND project may have been particularly useful for early career researchers who, as described by one respondent, were provided with a “unique scientific and networking opportunity.”

## 3.7. Scientific impact

### 3.7.1. EFPIA

**Figure 7:**
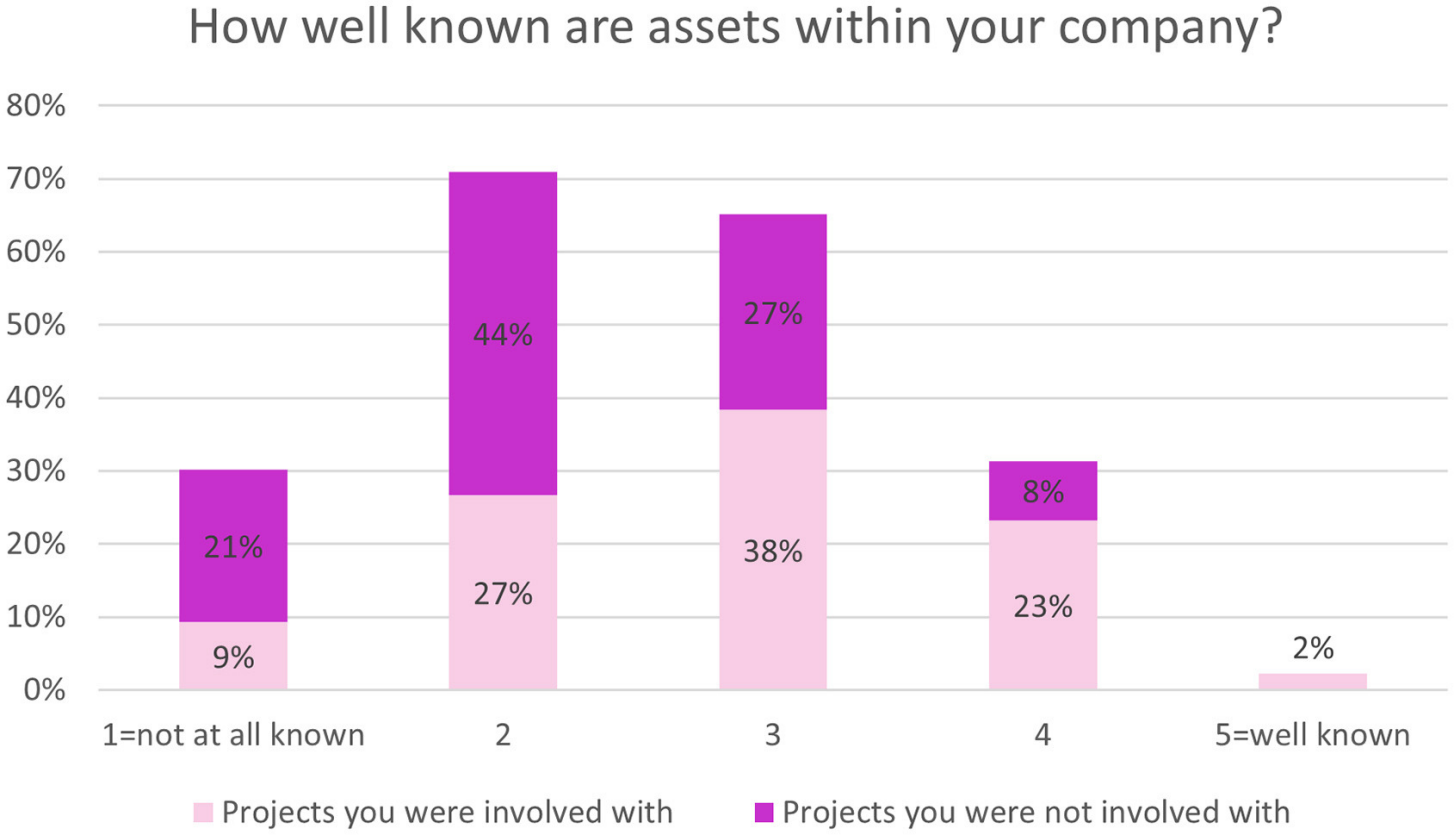
EFPIA survey questions assessing asset knowledge. Respondents were asked “how well are assets generated through IMI projects you were involved in, within your company?” (shown in light pink) and “how well are assets generated through IMI projects you were not involved in, within your company?” (shown in dark pink). Rating scale from 1 (not at all known) to 5 (well known). *N* = 86.

There was greater internal awareness of assets generated through IMI projects that respondents had been involved with compared to projects they were not involved with (Figure 7).

Respondents were not sure if assets were re-used within R&D (45%, *n* = 39/86) or if their company helped in sustaining project assets (69%, *n* = 59/86). Most respondents (53%, *n* = 46/86) were not sure if there is a central database within their company that contains information of assets generated in ND IMI projects, or whether the projects are changing the way that R&D is being conducted (42%, *n* = 36/86).

**Figure 8:**
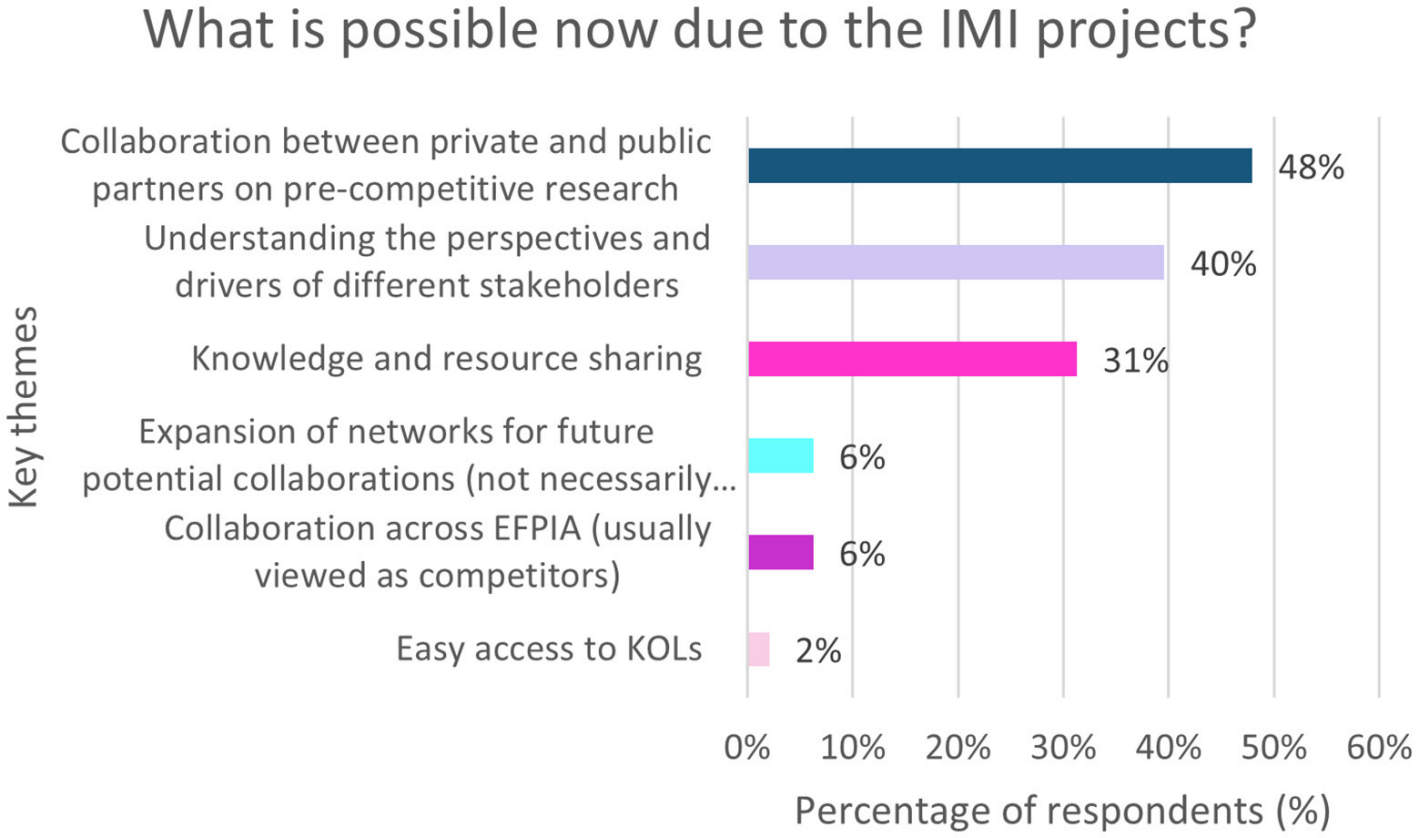
EFPIA survey question asking “what is possible now, that wasn't possible before these IMI projects?” This free text question was analyzed by theme which are summarized in this figure. *N* = 48 respondents who answered this question. KOL, Key Opinion Leader.

Overall, 56% (*n* = 48/86) of respondents provided insight into what is possible now, that was not possible before the IMI projects. The resulting key themes are shown in Figure 8.

### 3.7.2. Non-EFPIA

Most respondents (50%, *n* = 21/42) were unsure if results of the IMI ND projects had impacted the way science/drug development is being conducted. Of the remaining, equal proportions said “yes” and “no” (26%, *n* = 11/42 and 24%, *n* = 10/42, respectively).

Examples given of the changes to science/drug development due to the results of IMI projects included:

- Advances to and implementation of new technologies.
- More focused work e.g., focus on a digital biomarker, or greater focus on multiple targets.
- More rigorous processes.
- More integrated approaches.
- Higher level and more global thinking.
- Highlighted challenges in using multiple technologies with physically impaired samples.
- Project results will be used to inform future work.

The majority of respondents were aware of assets from other projects with 60% (*n* = 25/42) aware of “a few” and 14% (*n* = 6/42) aware of “many.” Only a quarter (26%, *n* = 11/42) of respondents were unaware of other projects' assets.

The majority (93%, *n* = 39/42) of respondents had not received requests for assets from other organizations.

## 3.8. Regulatory and policy impact

This was only assessed in the EFPIA survey.

Respondents were unsure if the results of the projects had an impact on regulatory practice (48%, *n* = 41/86) with similar proportions selecting yes and no (26%, *n* = 22/86 and 27%, *n* = 23/86, respectively).

## 3.9. Patient impact

### 3.9.1. EFPIA

This area asked whether the projects had brought science closer to patients and the general public. Of respondents 40% (*n* = 34/86) selected “yes.”

### 3.9.2. Non-EFPIA

Overall, similar proportions were either unsure (48%, *n* = 20/42), or believed (43%, *n* = 18/42) the IMI ND projects had successfully brought science and patients and the public closer together. One respondent noted that whilst this had not happened yet, there was a vision to do so once more solid results were available.

Those who felt that the IMI ND projects had brought science and patients and the public closer together, felt it did so through:

- Putting Patient and Public Involvement and Engagement (PPIE) at the core of activities, including study design and communication.
- Having high levels of contact with patients and patient representatives.
- Ongoing and wide dissemination of results.
- Outreach activities such as small group meetings, newsletters, conferences, public discussions and seminars.

## 3.10. Societal impact

### 3.10.1. EFPIA

In this section, respondents were asked if the general public and participants had been involved in the research, if it had given them a voice, better informed the public on ongoing research and results and paved the way for new patient-relevant treatment modalities. The most popular responses were “neutral” or “some impact” (37%, *n* = 32/86, and 35%, *n* = 30/86, respectively).

### 3.10.2. Non-EFPIA

Of non-EFPIA respondents, 78% (*n* = 33/42) reported either “moderate” or “high” impact when asked if the general public and participants had been involved in the research and if it had given them a voice.

## 3.11. Public health impact

### 3.11.1. EFPIA

In terms of impact on public health, 14% (*n* = 12/86) said yes, 55% (*n* = 47/86) selected “I don't know,” 31% (*n* = 27/86) said no.

### 3.11.2. Non-EFPIA

The majority of respondents (60%, *n* = 25/42) were unsure if the outputs from the IMI project(s) they worked on had an impact on public health, while 24% (*n* = 10/42) felt they did and 17% (*n* = 7/42) felt they did not. Two respondents thought that whilst they had not had an impact on public health yet, they would in the future.

Examples of impacts on public health reported by respondents included:

- Amyloid Positron Emission Tomography (PET) becoming routine in clinic.
- Possible new guidelines for application of digital health tools in mobility disorders.
- A new hypothesis based on IMI findings currently being tested clinically.
- Increased outreach, interest and knowledge including a number of peer-reviewed publications.

## 3.12. Advantages of involvement in IMI projects

### 3.12.1. EFPIA

**Figure 9:**
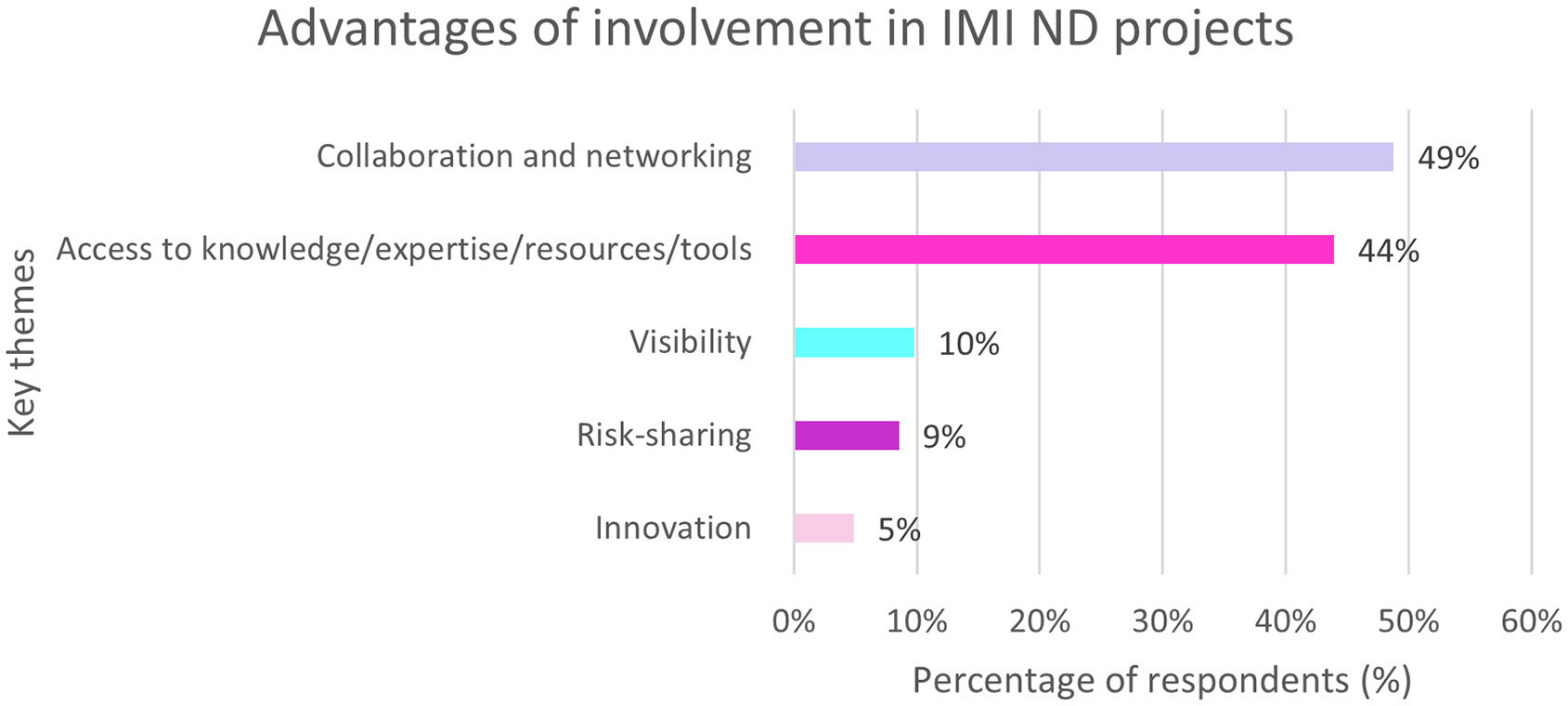
EFPIA survey question assessing the advantages of involvement in IMI ND projects. Respondents were asked to describe the main advantage of their company participating as EFPIA partner in IMI. The themes were analyzed and summarized in this figure. *N* = 82 respondents who answered this question.

Of the EFPIA respondents 95% (*n* = 82/86) felt that there were advantages associated with being involved in IMI ND projects. Almost half of respondents (49%, *n* = 40/82) cited “collaboration and networking” closely followed by “access to knowledge or expertise or resources or tools” (Figure 9).

Examples of advantages respondents gave were:

- “Acquiring and sharing knowledge and tools in a highly collaborative mindset.”
- “Improved networking; pre-competitive alignments and collaborations (reduce redundant R&D); boost company image for R&D.”
- “Discussions with experts in a specific field to solve rapidly existing experimental difficulties.”

### 3.12.2. Non-EFPIA

**Figure 10:**
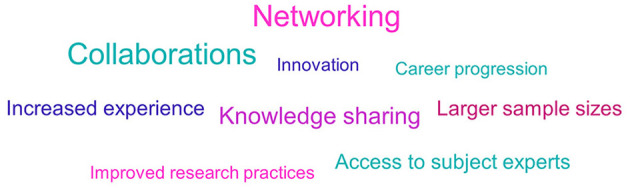
Non-EFPIA survey question assessing the advantages of involvement in IMI ND projects. Respondents were asked “from your experience, what were the main advantages and disadvantages of participating in an IMI project?” This figure shows the advantages, and the disadvantages are shown in Figure 12. The results were analyzed thematically. Larger font size indicates more frequent mentions. *N* = 33.

Opportunities for networking and collaborations was the most commonly cited advantage of being part of an IMI ND project (see Figure 10). Survey respondents stated that they welcomed the chance to build global relationships, have greater exposure to industry and regulatory bodies and strengthen intra-institute relationships. Another benefit was access to key opinion leaders.

A further area that respondents reported advantages in was within research practices and processes. Involvement in IMI projects was seen to provide access to larger sample sizes, image and data sets, and help improve research structures through sharing of best practice.

### 3.13. Disadvantages of involvement in IMI projects

#### 3.13.1. EFPIA

**Figure 11:**
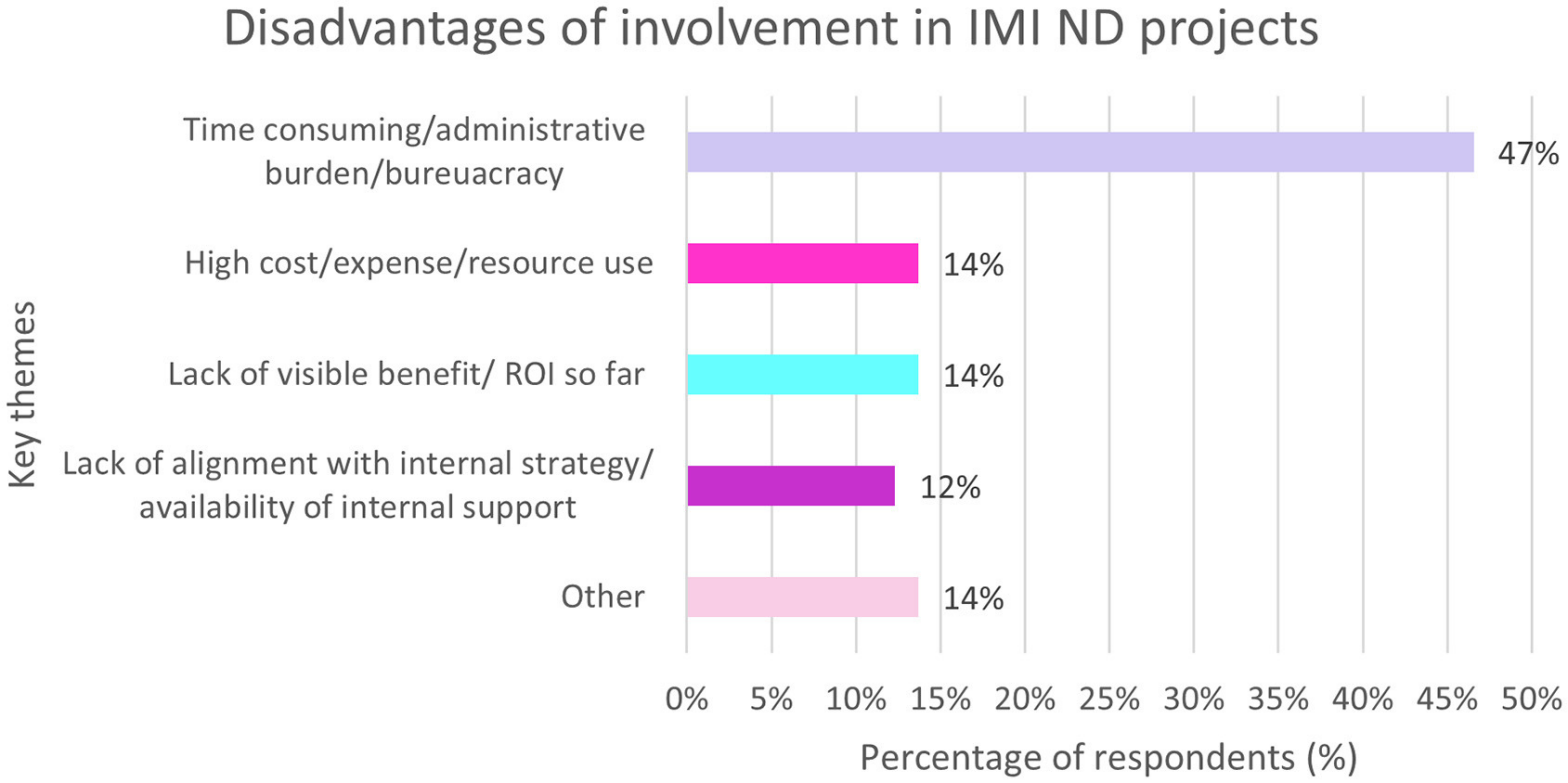
EFPIA survey question assessing the disadvantages of involvement in IMI ND projects. Respondents were asked to describe the main disadvantage of their company participating as EFPIA partner in IMI. The themes were analyzed and summarized in this figure. *N* = 62 respondents who answered this question.

Overall, 72% of responders reported disadvantages to being involved in the IMI projects. The most common was the time commitment and administrative burden. See Figure 11.

Examples of disadvantages respondents gave are:

- “Deviation of original plan due to continuous negotiation with public consortium leading to dilute results after 5 years.”
- “Requires more effort and time than initially thought it would take to positively contribute to the projects.”
- “Workload related to high documentation requirements.”

#### 3.13.2. Non-EFPIA

**Figure 12:**
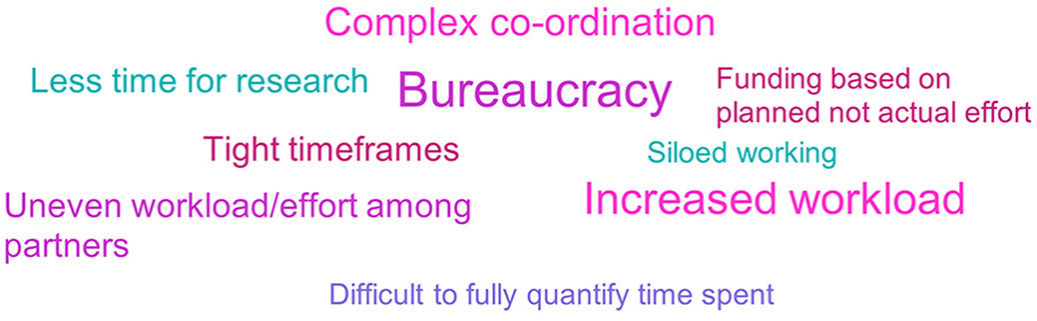
Non- EFPIA survey question assessing the disadvantages of involvement in IMI ND projects. Respondents were asked “from your experience, what were the main advantages and disadvantages of participating in an IMI project?” This figure shows the disadvantages, and the advantages are shown in Figure 10. The results were analyzed thematically. Larger font size indicates more frequent mentions. *N* = 33.

Only a small proportion of respondents (26%, *n* = 11/42) reported disadvantages of participating in an IMI ND project. Bureaucracy, increased workload and complex co-ordination were the most commonly cited disadvantages (see Figure 12). Respondents spoke of the large volume of additional administration required, including significant reporting requirements:

- “HUGE amount of reporting required by IMI, well-above and beyond other H2020 funding schemes.” Non-EFPIA survey respondent.

One respondent noted that the work was particularly demanding on SMEs, with no or very low profit.

Respondents felt co-ordination of projects was complex due to the large number of partners involved. Respondents reported that not only did co-ordination of projects require time and effort, but that at times it made delivery difficult because partners were not aligned. Tight timeframes added to this issue, and also made it hard to leverage learnings from data. While some respondents stated the large size of the consortium/projects as an advantage due to the experience and exposure it gave, others saw it as a disadvantage:

- “If too big, these projects become a series of silo projects. My experience with smaller IMI projects is much better than with larger ones”

A small number of respondents commented on what they considered uneven workloads within projects, and one noted the impact of funding allocation on this:

- “EPFIA contributions not clear or not very significant at times (Academic partners seem to be the most involved and put the majority of the effort).”
- “Some partners do much more to advance the project than others but this is not reflected in the amount of funding. So, when partners delay progress due to lack of effort, it is difficult to reallocate funding to a more motivated partner.”

## 4. Discussion

This study, conducted as part of the IMI NEURONET project, has shed light on the perceptions of project partners of the advantages and disadvantages of being involved in these public-private partnership projects. This is the first time this exploration has been undertaken systematically and across the two key stakeholder groups involved in these projects. Our results showed that the overwhelming advantages to being involved in IMI projects were the networking and collaborative aspects. This was true across both EFPIA and non-EFPIA respondents. It is not a surprising finding since the projects bring together people from different organizations. A bibliometric analysis of IMI research published between 2010 and 2021 found that two thirds of all IMI project papers were co-authored by researchers working in different sectors (12) which evidences the collaborative working. In addition, nearly all respondents reported meeting new people, both internally and externally. This advantage to working in the IMI ND portfolio has previously been cited (5, 13) and fostering radical collaboration between diverse public and private partners was found as a key success in a review of the IMI in 2019 (14). Our finding that the IMI ND projects are impactful in helping forge collaborations aligns with literature on this topic.

Unsurprisingly, the unanimous disadvantage to being involved in the projects was the burden of extra meetings, administration, increased workload and complex coordination. This was to be expected given the number and range of partners. Overall, respondents did not feel they had sufficient time to dedicate to the projects and a widespread comment was that even in cases where their manager or employer was supportive of their involvement, their normal workload was not adjusted. This is an area that should be considered in future projects and should be an important priority for funding bodies to address. This is not the first time this drawback has been documented (5, 14). This administrative burden is potentially jeopardizing the sustainability of interest in participating in these projects particularly for SMEs with limited head counts and administrative capacities and should be a key consideration going forward.

Both EFPIA and non-EFPIA respondents felt that involvement in the IMI ND projects had a clear impact on their organizations including strategic objectives and ways of working overall. However, awareness of assets and project outcomes was low among EFPIA respondents. Only 25% thought project assets were fairly well-known in their company and over half were unaware of a central database detailing project assets. This question assessed awareness of the NEURONET Knowledge Base (15) which had just expanded at the time of the survey. It is a platform that brings together key information and is designed to inform and facilitate similar new projects. This is not the first-time asset awareness has been found to be an area for improvement. This same recommendation was made by a group of experts tasked with evaluating phase one of IMI and performing an interim evaluation of the ongoing IMI2 initiative. One of their conclusions was that access to project outcomes should be broadened (16). Given the IMI objectives of speeding up drug development, it is essential for EFPIA companies to adopt the knowledge generated by projects, if the portfolio is to achieve impact.

Asset awareness did appear to be higher among non-EFPIA respondents. However, it may have been the phrasing of the question that led to this discrepancy. Non-EFPIA respondents were asked about their personal awareness of different assets whereas EFPIA respondents were asked about the awareness within their company. Asset awareness and sharing is a key success factor for PPPs (9). There are different models to achieve this. For example, The Division of Signal Transduction Therapy (DSTT) is a collaboration of six pharmaceutical companies and 20 academic research teams that share all unpublished results, along with reagents, technology and technical know-how. They credit this set up with causing them to publish more effectively (3) and the long-standing collaboration has led to the development and clinical approval of more than 40 drugs (3, 9). However, intellectual property (IP), and the incentives and laws surrounding this are a barrier to such transparent knowledge-sharing (7, 17) and a field of literature exists specifically looking at mechanisms to allow this whilst managing IP. This is arguably less of a concern in the pre-competitive space, which is where the IMI operates. On the basis of our findings, further research should be conducted to concretely determine whether asset awareness differs between these audiences. This would help develop appropriate and effective approaches to increase awareness.

A surprising result of the EFPIA survey was the conflict between whether IMI projects did or did not have an impact on R&D. When asked about changes to R&D through organizational impact the majority of respondents agreed that there were aspects done differently, and one respondent specified that a reduction in redundant R&D was an advantage to being involved in the project. Removing duplication of effort is a perceived advantage of PPPs (3). However, the results were reversed when asked a similar question as part of assessing scientific impact. A recent review demonstrates, with many examples, that PPPs in drug development and discovery do positively impact R&D (9) suggesting a positive impact on R&D is possible. It's important to establish if this survey finding is a true finding, or an artifact of the survey. A previous report examining the socio-economic impact of IMI1 projects specifically found that the projects were changing the manner in which new medicines are being developed, improving the R&D research infrastructure and streamlining the R&D (18). Impact on R&D is a key result considering the NEURONET objectives and further research should clarify if the combined IMI ND project portfolio is impacting R&D.

IMI ND projects are expected to facilitate, among other objectives, the development of new treatments and therefore provide patient impact. However, respondents' perception of this patient impact was uncertain and suggests that patient engagement in the projects might not be optimal. This notion is supported by a review of 75 IMI projects in 2017 which found that European or international patient associations were participating in only 16 of these projects (21%) (19). More could be done to include patients, ensure that the impact for patients is apparent and highlight the value of involving, engaging and communicating with patients at all stages of the pipeline. The authors of the review (19) identified 3 levels at which patient participation occurs: supporting with the dissemination of project results, providing a patient perspective from the start of the project or having a patient led project. The example of a patient-led project used by the authors was EUPATI which trained 100 patients in all aspects of medicine development and on developing an extensive, multi-language training toolbox to be rolled out across Europe (20). The levels of patient engagement outlined in the review could be a framework to increase patient involvement in IMI ND projects.

When the EFPIA respondents were asked about policy impact in terms of regulatory practice most stated they did not know. This could be linked to the types of projects that respondents were working on. The IMI ND projects span the whole pre-reimbursement pathway and whilst there are examples of projects focused on the HTA and regulatory end of the pipeline, many projects are pre-clinical and would not be expected to achieve a high impact on regulatory practice or public health. The long timelines in the life sciences and issues relating to translation of project results from bench to bedside are well-recognized challenges (5, 7, 14, 19), however IMI1 projects have been shown to result in downstream socio-economic impact (18). The review of publications also eludes to the projects having an impact at all stages of the product development timeline (12). This found that the IMI research was wide ranging from basic biological research to clinical practice. This suggests that whilst the projects in the IMI ND portfolio tend to operate upstream, they are likely to have impact further downstream including socio-economic impact and potentially impact on policy, regulatory and public health practice. Translating and aligning scientific results with regulatory requirements is a focus of IMI (21).

Overall, EFPIA staff reported spending less time on the IMI projects than the other partners involved. This could be expected as EFPIA partners provide financial input in addition to their staff time and may have multiple staff working on each project, therefore when surveyed each one reports less working time. It might be believed among non-EFPIA respondents that impact on career progression and opportunities is proportional to time spent on project tasks. This could also help in part explain why non-EFPIA respondents spent a greater number of weekly hours working on the projects.

### 4.1. Limitations

There were a few limitations in conducting this work. The original ambition was to directly compare responses from the EFPIA and non-EFPIA surveys to understand differences in perceived impact between the two audiences. However, in tailoring the non-EFPIA survey to be appropriate for the audience, the questions that assessed the same areas of impact had slightly different wording and this may have led to different interpretation in some areas. This meant it was only possible to make broad interpretations of the differences rather than direct comparisons. In addition, the adapting of the survey was done using assumptions by the working group about relevancy of themes for a non-EFPIA audience. Whilst this group had input from “non-EFPIA” members, it's possible non-EFPIA organizations could have provided insight into economic impact.

We conducted descriptive analyses but this does not allow conclusions about population parameters. A suggestion would be to perform inferential analyses with these survey data to allow conclusions about confidence, significance and any trends or correlations.

Other limitations relate to how extensively the areas of impact were explored. Lots of insight could be gleaned from the questions assessing the impact on the individual but only one question was asked to understand the impact on patients. Similarly, some questions could have fitted under multiple areas of impact. Finally, it was not possible to calculate a response rate because the number of those who received the survey was not recorded and recipients were asked to forward it to their relevant colleagues without the ability to track the number of recipients.

## 5. Conclusion

Overall, these surveys provided rich insight into the perceived impact of being involved in IMI ND projects. They revealed clear areas of impact and key advantages and disadvantages which were supported by the literature. Many were universal across both EFPIA and non-EFPIA audiences such as the benefit to collaborations and networking and the organizational impact. The unanimous disadvantage to being involved in the IMI projects was the extra administrative burden and time in meetings.

There were differences between the EFPIA and non-EFPIA respondents in terms of time spent on the project and asset awareness. Generally, the non-EFPIA respondents appeared to be more aware of project assets that had been generated. Further research should establish if this is a true difference to enable the design of appropriate communication strategies. More wide-spread access to the NEURONET Knowledge Base should help in growing the understanding and breadth of assets available. Further research should also clarify the impact of the IMI ND projects on R&D since this is a key area of impact and the survey gave mixed results.

Patient engagement and involvement is another area that requires focus. The survey indicated that respondents were uncertain of the patient impact and therefore more could be done to involve patients and highlight the value of including patients at every step of the project.

## Data availability statement

The raw data supporting the conclusions of this article will be made available by the authors, without undue reservation.

## Ethics statement

Ethical review and approval was not required for the study on human participants in accordance with the local legislation and institutional requirements. Written informed consent from the patients/participants or patients/participants' legal guardian/next of kin was not required to participate in this study in accordance with the national legislation and the institutional requirements.

## Author contributions

CH analyzed the data from the EFPIA survey and drafted the manuscript. FS developed and conducted the non-EFPIA survey along with LK who also developed the EFPIA survey and analyzed the data from the EFPIA survey. KC analyzed the non-EFPIA survey data with support from FS and CH. LP, DD, LS, and CD coordinated the project. All authors provided critical comments on the manuscript and approved the final version of the manuscript.
