# Supplementary material for: Participating in innovative medicines initiative funded neurodegenerative disorder projects—An impact analysis conducted as part of the NEURONET project
Source: Front Neurol. 2023 Mar 16;14:1140722. doi: 10.3389/fneur.2023.1140722 (PMC10060789; doi:10.3389/fneur.2023.1140722)
Supplement: Supplementary file 1 [file Table_1.DOCX]

Supplementary material

Table 1 (S1). The full list of 18 projects in the Innovative Medicines Initiative Neurodegenerative Disease Portfolio at the time of the survey, along with their start and end dates

|  | **Project** | **Start** | **End** |
| --- | --- | --- | --- |
| 1 | EMIF | 01/10/2013 | 30/06/2018 |
| 2 | AETIONOMY | 01/01/2014 | 31/12/2018 |
| 3 | EPAD | 01/01/2015 | 31/10/2020 |
| 4 | PRISM | 01/04/2016 | 30/09/2019 |
| 5 | RADAR-CNS | 01/04/2016 | 31/03/2021 |
| 6 | ADAPTED | 01/10/2016 | 30/09/2020 |
| 7 | MOPEAD | 01/10/2016 | 31/12/2019 |
| 8 | AMYPAD | 01/10/2016 | 30/09/2021 |
| 9 | PHAGO | 01/11/2016 | 31/10/2021 |
| 10 | ROADMAP | 01/11/2016 | 31/10/2018 |
| 11 | IMPRIND | 01/03/2017 | 31/01/2021 |
| 12 | EQIPD | 01/10/2017 | 30/09/2020 |
| 13 | RADAR-AD | 01/01/2019 | 30/06/2022 |
| 14 | IM2PACT | 01/01/2019 | 31/12/2023 |
| 15 | PD-mitoQUANT | 01/02/2019 | 31/01/2022 |
| 16 | Mobilise-D | 01/04/2019 | 31/03/2024 |
| 17 | PD-MIND | 01/05/2019 | 30/04/2022 |
| 18 | IDEA-FAST | 01/11/2019 | 30/04/2025 |

Table 2 (S2) The EFPIA survey

Table 3 (S3) The non-EFPIA survey

Table 4 (S4) The categorization of EFPIA and non-EFPIA survey questions into impact type

| **Type of impact** | **EFPIA survey questions** | **Non-EFPIA survey questions** |
| --- | --- | --- |
| Organizational | 6, 7, 8, 17, 18, 19, 26, 27 | 3, 4 |
| Economic | 9, 10 | - |
| Capacity building | 11, 12, 13, 14 | 4 |
| Collaborations, networks and partnerships | 15, 16, 39, 40, 41 | 11, 12, 13, 14, 18 |
| Individual | 29, 30, 31, 32, 33, 34, 35, 36, 37, 38 | 5, 6, 8, 9, 10 |
| Scientific | 20, 21, 22, 23, 24, 25, 28, 42, 45 | 16, 20, 21 |
| Regulatory and policy | 44 | - |
| Patient | 46 | 17 |
| Societal | 43 | 15 |
| Public health | 47 | 19 |

Table 5 (S5). The full list of European Federation of Pharmaceutical Industry and Association (EFPIA) companies represented by survey respondents. They are presented in descending order according to number of survey participants. For example, most survey respondents worked at Janssen.

| **EFPIA organizations** |
| --- |
| Janssen |
| Sanofi |
| Novartis |
| UCB |
| GE |
| Takeda |
| Pfizer |
| AC Immune |
| Bayer |
| Merck Kaa |
| Merck SDC |
| Servier |
| Icon |
| Psychogenics |
| Amgen |
| AZ |
| Biogen |
| Icon |
| Life |
| Lundbeck |
| Boehringer |
| Novo |
| Life |
| Grunenthal |
