# Supplementary material for: Participating in innovative medicines initiative funded neurodegenerative disorder projects—An impact analysis conducted as part of the NEURONET project
Source: Front Neurol. 2023 Mar 16;14:1140722. doi: 10.3389/fneur.2023.1140722 (PMC10060789; doi:10.3389/fneur.2023.1140722)
Supplement: Supplementary file 2 [file Data_Sheet_1.PDF]

IMPACT analysis- Academic and SME partners

Dear colleague,

Many thanks for taking the time to fill in this survey. The survey will take 10 mins of your time and your feedback will allow us to assess the impact of IMI neurodegeneration projects.

Involvement in Innovative Medicines Initiative (IMI) projects

1 Please select from below the option that describes your role

Question instructions: *Select one answer*

- ◯ Principal investigator
- ◯ Post-doctoral researcher
- ◯ Clinician
- ◯ PhD student
- ◯ Project manager
- ◯ Technician
- ◯ Other (please specify) [ ]

2 What proportion of your working hours do/did you spend on average on IMI neurodegeneration projects?

Question instructions: *Select one answer.*

- ◯ 5%-10% of my time
- ◯ 10%-50% of my time
- ◯ >50% of my time

Impact on research group or department and personnel

3 To what extent has your department changed (e.g. structure, size) as a result of its involvement in an IMI neurodegeneration project?

Question instructions: *Select one answer*

- ◯ Slightly
- ◯ Moderately
- ◯ Radically
- ◯ No impact

4 The involvement of your research group or company in IMI neurodegeneration projects has led to (tick all that apply)

Question instructions: *Select one or more answers*

- ☐ Expansion of current research lines
- ☐ Opening of new research lines
- ☐ Increase in the number of staff
- ☐ Improvement in your global positioning
- ☐ Result in new contracts or funding opportunities
- ☐ Other impact, please specify ____________

5 Did any new professional opportunities come your way directly/indirectly through participation in an IMI project?

Question instructions: *Select one answer*

- ◯ Yes
- ◯ No

6 Your involvement in IMI projects has had (choose one)

Question instructions: *Select one answer*

- ◯ A beneficial impact on your career progression
- ◯ A detrimental impact on your career progression
- ◯ No impact on your career progression

7 From your experience, what were the main advantages and disadvantages of participating in an IMI project?

____

Impact on research

8 Have you started working on any new products or with new research techniques as a result of participating in IMI project(s)

Question instructions: *Select one answer*

- ◯ No
- ◯ Yes, please explain ____________

9 Have you published any peer-reviewed publications based on your work in IMI project(s)?

Question instructions: *Select one answer*

○ Yes ○ No

10 Did you present any of your IMI project work at scientific conferences?

Question instructions: *Select one answer*

○ Yes ○ No

Impact on collaborations

11 Are there persons at YOUR organisation that you have newly met through working in IMI projects?

Question instructions: *Select one answer*

○ Yes ○ No

12 Are there persons at OTHER organisations that you have newly met through working in IMI projects?

Question instructions: *Select one answer*

○ Yes ○ No

13 Did these new collaborations result in

Question instructions: *Select one or more answers in each row*

	With an EFPIA partner	With an SME partner	With an Academic partner	There was no type of collaboration
Sharing of data, samples or materials	☐	☐	☐	☐
Joint publications	☐	☐	☐	☐
New joint research grant applications	☐	☐	☐	☐
Long-term scientific collaborations	☐	☐	☐	☐

14 Please provide details of other collaboration activities not covered in previous question

[ ]

Broader impacts on society, research and innovation

15 Rate the Societal impact (e.g. have the general public/participants more involved in research/give them a proper voice, inform public better on ongoing research/results of research, pave the way for new patient-relevant treatment modalities, etc) of IMI neurodegeneration research projects.

Question instructions: *1= No impact, 3=Neutral impact, 5=High impact*

☆☆☆☆☆ [ ] / 5

16 Did the results of IMI projects change the way science/R&D is being conducted?

Question instructions: *Select one answer*

- [ ] I don't know
- [ ] No
- [ ] Yes, please describe [ ]

17 Did these projects bring science closer to patients/general public and vice-versa?

Question instructions: *Select one answer*

- [ ] I don't know
- [ ] No
- [ ] Yes, please describe [ ]

18 Did you have an interaction with a regulatory or health technology assessment body in relation to your research?

Question instructions: *Select one answer*

- ○ No
- ○ Yes, please describe [ ]

19 Did outputs from IMI projects have a visible and directly measurable impact on public health?

Question instructions: *Select one answer*

- ◯ I don't know
- ◯ No
- ◯ Yes, please describe [ ]

Impact of assets

Table 1 below summarises the key assets from IMI projects, with the name of the project in bold and the corresponding project assets under it.

Table 1. List of assets produced by the IMI Neurodegenerative projects

**ADAPTED**
Multi-omics data from iPSC and ApoE mouse models
CSF and plasma -omics data from MCI patients
Biosamples from people with AD, MCI, or healthy individuals
iPSC-derived cell models of ApoE risk alleles
**AETIONOMY**
NeuroMMSig Server
Knowledge Base/ AData(Viewer)
Stratification algorithms
In silico model of neurodegenerative disease mechanisms
**AMYPAD**
Diagnostic and Prognostic study (DPMS)
Prognostic and Natural History study (PNHS)
Neuroimaging datasets from the AMYPAD PNHS and DPMS studies
**EBiSC2**
Disease relevant cohorts of hiPSC lines
Data on hiPSC lines and donors
Personal Data through DAC
HiPSC lines derived from different disease backgrounds
EBiSC public catalogue
EBiSC Data Access Committee
Human pluripotent stem cell registry (hPSCreg)
EBiSC template PIS and ICF
Protocols on use of hiPSC lines
hPSCreg nomenclature tools
Ethical and legal framework
Training videos on use of hiPSC lines

[illegible]
**EMIF-AD**
90+ study
Living systematic review
PreClinAD study
Clinical, neuroimaging and -omics datasets from EMIF-AD MBD, 90+ and PreClinAD studies
Blood and CSF from the EMIF-AD PreclinAD study
Blood samples, CSF and skin biopsies from the EMIF-AD 90+ study
Plasma, DNA and CSF from the EMIF-AD Multimodal biomarker discovery study (MBD)
Data Catalogue
Tools for federated EHR analysis
Risk factors for amyloid pathology, predictors for cognitive decline: clinical biology of AD
Procedures for federated data management
**EPAD**
Longitudinal Cohort Study (LCS)
Clinical, biomarker and neuroimaging data from the EPAD LCS study
Longitudinal Cohort Study biosamples
Trial delivery centre network
Register
Proof of Concept Trial Platform
Participant Registry in EPAD (PREPAD) tool
DerIDIOM tool
VElocity for EPAD (VEEPAD) tool
Subject Enrolment in EPAD (SEEPAD) tool
Research Participant Panel
Academy
**EQIPD**
Animal data from multi-site experiments
Tissue samples & DNA/RNA from in vivo and in vitro studies
Training Platform
Quality System
Variables in preclinical AD research that influence outcomes
Living systematic review
**IM2PACT**
-omics data on patient tissue and cellular disease models
In vitro and in silico models of the blood-brain barrier
**IMPRIND**
iPSC-based and organotypic cultures , neuronal models and animal models of alpha-synuclein or tau aggregation or propagation
Tools for isolating and characterising Tau & a-Synuclein, including aggregation assays
**MOPEAD**
Protocols for patient engagement
**PHAGO**

[illegible]
Neuroimaging data from the KCL neuroimaging study
CSF samples from TREM2 cohort KCL
Data and Knowledge Platform from Fraunhofer
Tools and assays for targeting and analysing TREM2 & CD33
**RADAR-AD**
Real-world data from RADAR-AD study of multiple wearable and digital devices
Patient Advisory Board
Advanced augmented reality monitor for assessing the risk of Alzheimer and Dementia
Advanced data management dashboard for monitoring of data continuity from remote sensors
Advanced passive remote monitoring (pRMT) application for user behavior modelling.
Daily life tasks monitoring app for cognitive status assessment (Banking App)
Manual data upload tool for remote monitoring sensors data acquisition files.
Mezzurio - A smartphone app supporting active participation in research studies and clinical trials
**RADAR-CNS**
MS Cohort
Epilepsy Cohort
Depression Cohort
Radar Base platform
Patient Advisory Board
**ROADMAP**
Data cube
Relevant functional outcomes for different stakeholder groups
EXAG Advisory Agreement Template

20 Looking at the summary of assets produced by IMI projects in Table 1 above, could you please indicate if you are aware of

Question instructions: *Select one answer*

○ Only assets from your project ○ A few assets from other projects ○ Many assets from other projects

21 Have you received requests for assets from other organisations?

Question instructions: *If yes, please provide details*

○ No

○ Yes, please provide details [ ]
