# Supplementary material for: Participating in innovative medicines initiative funded neurodegenerative disorder projects—An impact analysis conducted as part of the NEURONET project
Source: Front Neurol. 2023 Mar 16;14:1140722. doi: 10.3389/fneur.2023.1140722 (PMC10060789; doi:10.3389/fneur.2023.1140722)
Supplement: Supplementary file 3 [file Data_Sheet_2.PDF]

IMPACT analysis EFPIA

Questions 1-5 are about your experience in Innovative Medicines Initiative (IMI)

1 How many Neuroscience IMI Projects have you personally participated in (past and ongoing Projects)?

2 In how many Neuroscience IMI Projects have you personally been involved as Project Leader?

3 In how many Neuroscience IMI Projects have you personally been involved as Work Package Lead?

4 In how many Neuroscience IMI Projects have you personally been involved as Task Lead?

5 How much of your time did you work on Neuroscience IMI projects on average (number of hours/ week)?

Question instructions: *Select one answer*

- [ ] Less than 2 hours per week
- [ ] 2-5 hours per week
- [ ] 6-15 hours per week
- [ ] If other, please specify

Questions 6 up to 28 are about the **IMPACT on Company. Please answer these questions by sharing your own perspective.**

6 Rate impact on the company's and/or Therapeutic area (TA) Strategic Objectives and way of working overall: Did the IMI projects you were involved in contribute to achieving Strategic Objectives or influence these (e.g. faster compound development, data sets and tools developed, expanding to new research areas/modalities etc.)? Did IMI change or influence the way the company is operating (e.g. increased external collaborations, co-development opportunities, collaborative/IIS studies, etc.)?

Question instructions: *1= No Impact, 3= Neutral, 5= High Impact*

☆☆☆☆☆ ☐ / 5

7 Are there any aspects of R&D we do differently because of these projects (e.g. having a stronger patient voice, more research through external collaborations, use of certain tools/methodologies/algorithms, knowledge that was internalised)?

Question instructions: *Select one answer*

- [ ] I don't know
- [ ] No
- [ ] Yes - Please describe

8 Rate the impact on Company's presence/visibility/public perception (e.g. Did these projects contribute to a positive company image? Did it improve the image of the company as a trustworthy partner?)

Question instructions: *1= No Impact, 3= Neutral, 5 = High Impact*

☆☆☆☆☆ ☐ / 5

9 Rate the return on investment in terms of increased efficiency, acceleration of processes, new knowledge, etc.

Question instructions: *1= No Impact, 3= Neutral, 5= High Impact*

☆☆☆☆☆ ☐ / 5

10 If possible, could you elaborate on what project outcomes trigger the return on investment (ROI)?

11 Rate the impact on attracting new talent.

Question instructions: *1= No Impact, 3= Neutral, 5= High Impact*

☆☆☆☆☆ ☐ / 5

12 How many persons (e.g. post-docs) did the company hire specifically to work on an Neuroscience IMI Project you have worked on?

13 How many of these persons received a permanent position within the company, either during the Neuroscience IMI project or after completion of the Neuroscience IMI project?

14 How many persons did the company hire through IMI projects, who were initially working on a Neuroscience IMI Project affiliated to another Project Partner?

15 Rate the impact on establishment of strategic partnerships.

Question instructions: *1= No Impact, 3= Neutral; 5= High Impact*

☆☆☆☆☆ ☐ / 5

16 Are there any strategic partnerships formed between the company and other IMI partners? E.g. Academic partners/SME (small and medium-sized enterprises) /European Federation of Pharmaceutical Industries (EFPIA partners)?

Question instructions: *Select one answer*

☐ I don't know

☐ No

☐ Yes - Please describe

17 Describe the main advantage of your company participating as EFPIA partner in IMI.

18 Describe the main disadvantage of your company participating as EFPIA partner in IMI.

19 How well known is IMI within your company?

Question instructions: *1= Not at all known, 5 = Well Known*

☆☆☆☆☆ ☐ / 5

20 How well known are assets (e.g. tools, datasets...) generated through Neuroscience IMI projects you were involved in, within your company?

Question instructions: *1= Not at all known, 5 = Well Known*

☆☆☆☆☆ ☐ / 5

21 Briefly describe a couple of assets if any (relates to the previous question)

☐

22 How well known are assets (e.g. tools, datasets...) generated through Neuroscience IMI projects you were not involved in, within your company?

Question instructions: *1= Not at all known, 5= Well known*

☆☆☆☆☆ ☐ / 5

23 Briefly describe a couple of assets if any (relates to the previous question)

☐

24 Are you aware of any (re)use of the assets in R&D?

Question instructions: *Select one answer*

☐ I don't know ☐ No

☐ Yes - Please describe ☐

25 Does your company help in sustaining project assets from Neuroscience IMI Projects?

Question instructions: *Select one answer*

- [ ] I don't know
- [ ] No
- [ ] Yes- Please describe

26 Does your company help in creating awareness on (outcomes of) Neuroscience IMI projects?

Question instructions: *Select one answer*

- [ ] I don't know
- [ ] No
- [ ] Yes- Please describe

27 Does your company help in creating awareness on the impact of (outcomes of) Neuroscience IMI projects?*

Question instructions: *Select one answer*

- [ ] I don't know
- [ ] No
- [ ] Yes- Please describe

28 Is there a central database or knowledge base within your company that contains (descriptive) information of assets generated in Neuroscience IMI projects?

Question instructions: *Select one answer*

- [ ] I don't know
- [ ] No
- [ ] Yes- Please describe

Questions 29-35 are about the IMPACT on your daily work

29 Rate the impact of IMI on how you perform your daily tasks

Question instructions: *1= No Impact, 3= Neutral, 5= High Impact*

☆☆☆☆☆ [ ] / 5

30 Are there tasks you do differently?

Question instructions: *Select one answer*

- [ ] I don't know
- [ ] No
- [ ] Yes- Please describe

31 Are there new tools/datasets/knowledge you use for your daily work (created/known via an IMI project)?

Question instructions: *Select one answer*

- [ ] I don't know
- [ ] No
- [ ] Yes- Please describe

32 Any impact on your daily tasks by participation in Neuroscience IMI projects, you want to highlight?

33 Did you get any support from your employer (supervisor) for your assigned tasks in these Neuroscience IMI projects?

Question instructions: *Select one answer*

- [ ] Yes
- [ ] No
- [ ] If other, please specify

34 Did you receive any appreciation from your employer (supervisor) by working on these Neuroscience IMI projects?

Question instructions: *Select one answer*

- [ ] Yes
- [ ] No
- [ ] If other, please specify

35 Did you have sufficient resources/time to fulfill your assigned tasks in these Neuroscience IMI projects?

Question instructions: *Select one answer*

- [ ] Yes
- [ ] No, mostly on top of my daily activities
- [ ] If other, please specify ______

Question 36-38 are about the IMPACT on your professional career

36 Describe how IMI has improved/impacted your skillset.

______

37 Did participation in IMI expand your (scientific) horizon?

Question instructions: *Select one answer*

- [ ] I don't know
- [ ] No
- [ ] Yes- Please describe ______

38 Did any new opportunities come your way directly/indirectly through participation in an IMI project? Any personal development opportunities as a result?

Question instructions: *Select one answer*

- [ ] I don't know
- [ ] No
- [ ] Yes- Please describe ______

Questions 39-41 are about the IMPACT on your professional network

39 Are there persons at your own company that you have newly met (e.g. from other departments, divisions) through working in IMI projects?

Question instructions: *Select one answer*

☐ 0 ☐ 1-5 ☐ 6-10 ☐ 11-15

☐ If other, please specify [ ]

40 Are there persons at other companies that you have newly met through working in IMI projects?

Question instructions: *Select one answer*

☐ 0 ☐ 1-5 ☐ 6-10 ☐ 11-15

☐ If other, please specify [ ]

41 Are there new longterm relationships with Academic Institutions/ SMEs/ Biotechs/ Patient Organisations/ Regulators/... (long term alliances, enacted collaborations, more possibilities to outsource) you've established through working in IMI projects?

Question instructions: *Select one answer*

☐ 0 ☐ 1-5 ☐ 6-10 ☐ 11-15

☐ If other, please specify [ ]

Questions 42- 47 are about the IMPACT on the field at large

42 What is possible now, that wasn't possible before these IMI projects?

[ ]

43 Rate the Societal impact (e.g. have the general public/participants more involved in research/give them a proper voice, inform public better on ongoing research/results of research, pave the way for new patient-relevant treatment modalities, etc)

Question instructions: *1= No Impact, 3= Neutral, 5= High Impact*

☆☆☆☆☆ ☐ / 5

44 Did results have an impact on regulatory practice?

Question instructions: *Select one answer*

☐ I don't know ☐ No

☐ Yes- Please describe ☐

45 Did results change the way science/R&D is being conducted?

Question instructions: *Select one answer*

☐ I don't know ☐ No

☐ Yes- Please describe ☐

46 Did these projects bring science closer to patients/general public and vice-versa?

Question instructions: *Select one answer*

☐ I don't know ☐ No

☐ Yes- Please describe ☐

47 Did outcomes have a visible and directly measurable impact on public health?

Question instructions: *Select one answer*

☐ I don't know ☐ No

☐ Yes- Please describe ☐
